# Addressing the challenges facing the paramedic profession in the United Kingdom

**DOI:** 10.1093/bmb/ldad024

**Published:** 2023-09-07

**Authors:** Georgette Eaton

**Affiliations:** Nuffield Department of Primary Care Health Sciences, University of Oxford, Radcliffe Observatory Quarter, Woodstock Road, Oxford OX2 6GG, UK

**Keywords:** paramedics, ambulance services, professional identity

## Abstract

**Background:**

The paramedic profession within the United Kingdom has been evolving at pace over the last 20 years. While they are most associated with their work in ambulance services, paramedics are now found throughout a range of clinical and academic settings.

**Sources of data:**

Literature Review.

**Areas of agreement:**

Despite emergence of the paramedic across the healthcare workforce, the understanding and awareness of the professional role and capabilities is poor. This could be due to a lack of representation within senior leadership roles and within health and social care policy.

**Areas of controversy:**

Understanding of the paramedic professional identity, from a philosophical and sociological perspective, remains incomplete.

**Growing points:**

A challenge for the future is to explore how the paramedic role can continue to develop across a range of clinical settings, while retaining its *sui generis* professional identity.

**Areas timely for developing research:**

Research to establish perspectives of and about the paramedic profession may assist in developing an understanding of identity and its place within the wider healthcare workforce.

## Background

It is the Greek word πᾰρά that gives the English prefix of ‘para’, meaning alongside, besides or near.^1^ Inherent in the professions title is their work alongside medicine. The crucial element here is alongside. Not as an auxiliary, assistant or associate—but in parallel, autonomous. This correlates with a profession who practices without direct medical oversight, for whom the delivery of urgent and emergency care in the out-of-hospital environment is their *raison d’etre*, and for what they are most commonly associated with.

However, paramedics are one of the most rapidly developing professions in the United Kingdom (UK). Since 2000, paramedics have been required to hold professional registration, and the title subsequently protected in law though Article 39^1^ of the Health Professions Order 2001. While undergraduate honor’s degrees have only recently been mandated for paramedics to enter the state register,^2^ the first paramedics entered into the academy some 30 years earlier, completing undergraduate degrees at the University of Hertfordshire.^3^ The last decade has seen the profession achieve the ability to undertake independent prescribing, as well as a more prominent expansion into other clinical settings, including primary care, end-of-life care and tentative steps into highly specialized roles such as neonatal care. This is alongside the profession governing their professional education,^3^ developing the professional research agenda^4^ and, more recently, obtaining leadership appointments as directors of paramedic services.^5^

Yet, this high growth profession is not without its challenges.

## Paramedics and the evolution of ambulance services

Within the UK, the concept of a paramedic (as a registered profession with a title protected in law) has recently entered its twentieth decade, but the history of the paramedic is considerably older than that. As the name would suggest, paramedics have been found working alongside physicians since their earliest debut, commonly accepted to be during the Crusades of the 11th Century. At this time, the Order of the Knights Hospitaller received instruction by physicians in order to provide remote emergency care to treat soldiers injured during the battles, as well as providing care to sick pilgrims. Such models in physician devolution in the provision of care have continued since, re-emerging in wars across the globe until making its way into civilian practice in the South of England in the early 1970s.

Paramedics continue to be most commonly associated with their work in ambulance services, which typically operate a delivery model of care: Responding to a call, initiating treatment and transporting the patient to definitive care. However, many ambulance services in the UK now position themselves as mobile health care providers in addition to providing the emergency medical care stereotypically associated with them. Moreover, rather than merely providing patient transportation to the nearest emergency department, they are equipped to provide definitive care at the location of the call, either through remote hear-and-treat services or through on-scene assessment. The necessity to develop ambulance services (and the paramedics therein) has come from changes in their call demand, with just 8% of calls to 999 from people with life-threatening injuries or illness.^6^ Attending to minor injury and illness through the 999-call system are now much more commonplace, as are calls relating to chronic disease management, mental health crises and social care needs^7^—all of which place a significant burden on ambulance service response.^8^ While much of this demand encountered by ambulance services could be handled by a range of other healthcare providers, including primary care services, community nursing services or specialist medical departments who have primacy of patient management, these options are either not available throughout the 24-h period or, where they are, do not always have the resources to deliver the level of responsiveness expected by the public. In many cases, the accessibility of calling 999 and the responsiveness of the ambulance services is considered to act partly as a safety net for gaps (and failures) in other parts of the National Health Service (NHS).^9^

Yet, despite this change in call type, paramedics in the ambulance service are still expected to be able to perform advanced life support and the emergency procedural skills that have, in part, characterized the profession—despite the fact the need for such skills accounts for ˂10% of their contemporary workload. These changes in demand, demographics and epidemiology have led some researchers to conclude that paramedicine and the UK ambulance service is moving from the ‘extreme to the mundane’.^10^

## Paramedics in the wider clinical workforce

It is perhaps this interplay between extreme and ‘mundane’ that equips paramedics to practise elsewhere. In the ambulance service, expecting the unexpected is the *modus operandi* of the workforce, and it is perhaps this that makes paramedics suitable to work in other clinical settings which do not have the luxury of point of care testing or imaging, where reliance is on the clinical acumen, clinical gestalt and clinical courage of its practitioners. It stands to reason that paramedics are well-acquainted not only in working in austere environments or remote communities, but also in developing risk management models as they navigate challenging decisions in the out-of-hospital setting.^11^

The ‘mundane’ elements of work in ambulance services may have also enabled paramedics to develop as generalist clinicians. Indeed, paramedics who see themselves as generalists seek opportunities to work in primary care, believing their capabilities would fit well within this workforce.^12^ While popularized in current workforce planning,^13^ it is not only primary care that paramedics may seek to work in, away from the ambulance service. The development of a multidisciplinary workforce in the emergency department has resulted in paramedics also moving into this clinical environment, predominantly working in an Advanced Clinical Practitioner role with the opportunity to credential with the Royal College of Emergency Medicine.^14^ In more recent years, paramedics have been eligible to work in Surgical Care Practitioner posts, and have been documented working in hospice settings,^15^ providing health promotion advice,^16^ playing a role in identifying modern slavery,^17^ as well as in highly specialized roles such as neonatal transfer services.^18^

While there are examples of paramedics moving into other clinical settings, what is not known is the number of paramedics in these clinical settings. This problem is articulated particularly well in primary care where, despite the number of paramedics who are estimated to be working there, national workforce surveillance data has only recently captured the paramedic job role—and even then, many paramedics are reported locally under ‘nurse’ or generic healthcare professional roles, and then affects the census data captured from national database systems.

It is in part due to their mobility across the health workforce that has resulted in paramedics (as well as other AHPs, nurses and physician associates) being referred to as ‘noctors, phoctors and mocktors’.^19^ Though there can be no replacement for physicians, NHS reforms over the past decade have been aimed at broadening the scope of the healthcare workforce to make it more generic with the goal of increasing flexibility to cope with the changing healthcare needs of the population—and this is where advanced practice has come into its own. The pluripotent generalist paramedic fits in well with such reform. However, coupled with the ongoing issues relating to retention of physicians in community and hospital settings^20^ and ongoing disputes regarding pay, it is unsurprising that non-physician roles are met with a mixture of alarm, concern or skepticism by some physicians. What critics of the flattening of this hierarchy fail to realize is that the allied health professions and nurses whose professional roles have expanded as a result of these reforms are still regulated professions, and who are accountable for their practice. This lack of knowledge and awareness of the profession in terms of its capabilities presents a challenge for paramedics entering clinical settings away from the ambulance service.

The UK offers perhaps the broadest palette of roles for paramedics beyond the traditional out-of-hospital role and this expansion of opportunity has been welcomed by the profession. However, it does raise issues as to whether those who perform the roles traditionally occupied by nurses or physicians are still paramedics, and how educationally prepared they are to undertake such roles.

## Changes in the provision of paramedic education

Paramedic education previously focussed on the provision of trauma, resuscitation and procedural skills training for paramedics to respond to time-critical events. However, as the changing case load to respond to 999 calls has changed, so has the content of the paramedic curriculum. There can be no doubt that this broad and undifferentiated case mix has been responsible, in part, for encouraging undergraduate education to become the entry point to the profession, and the foundation to enable paramedics to move into other clinical settings.

The current paramedic undergraduate curriculum^3^ has caught up with the changing case mix experienced in the ambulance service and today’s paramedic degree programs oftentimes have placements in primary care, rehabilitation units and urgent care services. However, the taught components now may not always match the experience of paramedic educators in the academy—who, if they have not kept currency with clinical practice, will not have contemporary clinical experience with which to draw on and teach.^21^

Another challenge faced by paramedic educationalists is the lack of standardization in the delivery of undergraduate degrees across the UK. While each education provider must be approved by the Health and Care Professions Council (the regulatory body for paramedics), endorsement by the College of Paramedics for a program that follows their curriculum guidance is not mandatory. The HCPC approvals process focusses on determining whether an institution meets the education standards at the threshold level required, so that students become eligible to register and demonstrate the expected Standards of Proficiency. This is not in place of a curriculum, which sets out the essential concepts to prepare students to undertake the role on registration.

Yet no undergraduate curriculum could reasonably hope to capture such range of clinical opportunity available for the profession in the 21st Century. While the current curriculum gives preparation for the student paramedic in the assessment and management of an undifferentiated patient group (in urgent and emergency case presentations), much of the learning for this practice profession would be experiential, in clinical placements during the degree, or the initial years post registration. This is not too dissimilar to medicine. However, while foundation years of structured support and development are the backbone of medical education, such concepts for paramedics (and other allied health professionals) are only now being introduced.^22^ During their formative years, paramedics have notoriously lacked the clinical supervision and meaningful feedback that has benefitted medical training, and it is such poor experiences in these foundation years that have resulted in graduates leaving ambulance services,^23^ and potentially the profession. Although the concept is there, such programs still need to be developed for newly qualified paramedics to benefit from them, and it will be of interest to many to see whether these will limit paramedics to the ambulance service in the first 2 years post-registration or be open across the range of clinical settings paramedics are currently found practising in. Currently, the lack of standardization of preceptorship programs results in a lack of direction for newly qualified paramedics.

Standardization is afforded, however, with the advent of Advanced Clinical Practice across the UK, where guidance is offered for development of paramedics (alongside other allied health professionals and nurses) to practise at this advanced level following completion of postgraduate study.^24–27^ Frameworks across the devolved nations each set out the standards expected of Advanced Clinical Practitioners, the core capabilities required for the eligible professions, and the routes available to this level of practice—which is typically after 5 years clinical experience and a Master’s Degree in the subject. This fosters far more opportunity for standardization than is currently afforded by the education facilitating entry to the paramedic register. However, it also results in a generic healthcare workforce, lacking the *sui generis* of the individual professions that may come to advanced practice.

### Paramedic independent prescribing

Associated with postgraduate study is also the ability to undertake independent prescribing, arguably the biggest clinical development for the profession since its initial inception. Legislated in 2018, paramedics are able to prescribe within the scope of role in the area in which they work, though this is often subject to local or organizational formularies. For non-controlled drug medication, the only restriction on practice is that a paramedic prescriber cannot prescribe unlicensed medication. However, perhaps the most significant single restriction in current paramedic prescribing practice is the lack of the ability to independently prescribe any controlled drug. While the Human Medicines Regulations 2012 detail paramedics new authority to prescribe non-CDs, the legalities of prescribing CDs fall under the Misuse of Drugs Act 2001 that has not yet changed. It is perhaps because the number of paramedic prescribers is still relatively small, and so they lack the voice within institutional structures to lobby for change in the law (though it is said to be on the horizon).^28^

For the most part, paramedic independent prescribers work in primary care, emergency departments and hospice settings^29^—clinical environments where the prescription of controlled drugs such as opiates and benzodiazepines are commonplace. Thankfully, in emergency situations (where the need for the autonomous use of these medicines is arguably greater) paramedics can administer these drugs under exemptions listed in the Human Medicines Regulations 2012. However, this does little to treat the patient with back pain who presents to the paramedic in primary or urgent care—and it is in such situations that result in duplication of contact for the patient, and potential increase in workload for General Practitioners (who ultimately need to see and treat the patient).^12^

## A crises of paramedic identity

Perhaps by their *de novo* design and subsequent evolution, paramedics are pluripotent—capable of development in several different ways. This fits in well with the aim of advanced practice for experienced and highly educated clinicians. Indeed, the more skilled a paramedic becomes in their clinical care, the more autonomy the paramedic has—which is something that paramedics value.^30^ However, as they gain in experience and skill, paramedics are more likely to work in roles beyond what is considered the traditional paramedic role—as demonstrated with advanced practice in primary or emergency care, or in highly specialized roles such as neonatal transfer practitioners. This risks the skills and role of the paramedic becoming increasingly generic, which in turn results in paramedics being less clear about the limits of their professional responsibilities and professional identity.^31^ There is of course great merit in expanding skills, and offering clinical development across the profession, but it is likely to have unintended outcomes of reshaping paramedic professional identity.

Maybe this generalization and broadening of roles enables us to better understand the true nature of the paramedic. Let us first consider the professional title, protected in law: The Greek word πᾰρά gives the English prefix of ‘para’, meaning alongside,^1^ and ‘medic’ clearly relates to medicine. While autonomous professionals in their own right, paramedics operate *alongside* medicine, not independent of it, nor combined with it. It is this element of the profession that most aptly enables the diversification of the paramedic profession: For (to a great extent) where medicine is practised, so too can paramedicine. This nomenclature serves to differentiate between the paramedic and the physician associate. While both may work in the same clinical settings, with the potential to fulfill similar clinical roles, the latter verb ‘associate’ being ‘*to join, combine in action, unite’*^32^ clearly differentiates the role of the two professions regarding independence and responsibility in healthcare. Paramedics are autonomous at the point of registration, but integrated working is a key component of paramedic practice. Across all clinical settings, paramedics also work closely with other health and social care professionals, such as (but not limited to) General Practitioners, pharmacists, medical and surgical consultants, nurses, social care providers—as well as police, fire and rescue services and the coastguard when as part of an emergency medical service. Such range of opportunity for integrated working, while retaining autonomy, is unique to paramedics, and a core component of their professional standards.^33^

The benefits of diversification have arguably enabled paramedics to refresh their identity and understand their place in the wider healthcare workforce. Remaining alongside medicine, they have demonstrated a broad and increasing functionality. Such utility has an impact on socio-political status and power; moving from a ‘blue-collared’ ambulance-based occupation^34^ to a recognized, autonomous, profession further compounds the attractiveness of paramedics to work across a range of clinical domains.

As the healthcare workforce diversifies, does it matter if these roles are generic, rather than distinct in some way from other healthcare practitioners? Sociologists like Freidson^35^ would undoubtedly say yes, as professions are shaped by their unique body of skills and thus contribution to the workforce. Yet, it is not possible to understand the distinctiveness of paramedic identity just by examining what paramedics ‘do’ in relation to their skills, capabilities or contribution to the wider workforce. In noting this, of course, there are some roles and procedures that paramedics perform more commonly and predominately than other professional groups, and they do so in a very different environment. Understanding not just what paramedics ‘do’ but where it is undertaken contributes to the profession ‘knowing’ themselves—and this point of distinction should not be dismissed in trying to define paramedic identity. Indeed, Freidson suggests that some professionals ‘*do not merely exercise a complex skill, but identify themselves with it’*. Formative learning in the austere ambulance environment during their undergraduate degree embeds the professional approach to ‘doing’ and it is through this that one ‘becomes’ a paramedic. The uniqueness of the profession needs to integrate aspects of ‘doing, knowing, being and becoming’^36^ if it has any hope of retaining its *sui generis* identity. In defining the paramedic identity, it would be wrong of the paramedic profession to ignore its origins as distinct and unique providers of out of hospital emergency care that have evolved over time, and the paramedic identity can, and must, continue to evolve in this occupational domain.^37^ However, for those paramedics who work elsewhere, it will be critical that they reflect on what makes their work distinct from the others they work with—and how they retain their professional identity in light of this diversified workforce.

Contemporary paramedics are evidently much more than the ‘ambulance drivers’ they perhaps once were. However, what has become apparent is the lack of representation for the profession in policy and national leadership roles. Paramedics have no national clinical advisor within NHS England, despite similar roles existing for Nursing,^38^ pharmacists^39^ and therapy/rehabilitation professions.^40^ This lack of advocacy for the profession also hinders the ability to establish the value and highlight the importance of what professions can do, as well as tackling embedded stereotypes that surround the role. While paramedics are anecdotally listed as one of the most trusted professions (ranking fourth after doctor, nurse and teacher),^41^ there is currently no empirical UK evidence that explores social attitudes toward the profession. The paramedic professional body, the College of Paramedics, is seeking royal college status in an effort to increase public confidence and awareness of the expertise of the profession. If this application is approved, it could potentially lead to an elevation in social prestige for the paramedic profession.

## International considerations

One particular challenge facing the UK paramedic profession is the global position of the profession. In part, the development of the paramedic profession across the globe is characterized by the primary model of emergency medical service implemented within each country. Historically, emergency healthcare delivery in a pre-hospital environment has revolved around two models: The Franco-German model follows the ‘stay and stabilise’ stance, where prehospital physicians and para-medical staff bring the hospital to the patient, operating as a sub-set of the wider health system. In contrast, the Anglo-American model operates on a ‘scoop and run’ approach, prioritizing swift patient transportation by trained emergency service workers to an emergency department for definitive assessment treatment.^42^ One could make the case that the widespread adoption of the physician-led Franco-German model throughout mainland Europe has potentially hindered the growth of para-medical staff as an independent profession across much of this continent. Similarly, the limited demand for the Anglo-American model (primarily seen in the United States of America) to deliver definitive treatment might reasonably explain the requirement for firefighter-paramedics or emergency medical technicians-paramedics within these systems to undergo vocational training exclusively.

While today, most emergency medical systems will combine elements of both seminal models, the impact of these on the subsequent development of the paramedic profession within that model should not be underestimated. It is therefore unsurprising that paramedicine has developed differently between different countries, and even between different states and provinces (as in the USA and Canada). As well as the operational of the role, these differences are also incurred in the nomenclature associated with the role, such as ‘rescuer’ or ‘technician’. This disparate state does little to support professionalization as a global profession, which is in direct contrast to medicine and nursing, for which exist globally accepted standards of education, and consequently the option for professional practice internationally.^43^^,^^44^ The lack of globally recognized education and associated professional standards, as well as disparity between regulatory functions across different countries and continents, mean that paramedicine is not afforded the same global mobility as their physician and nursing counterparts. That is not to say that a paramedic from the UK cannot work in Canada (for example), but that the variation in how the profession has developed and subsequently been operationalized does not support mobility on a global scale. This discrepancy also prevents paramedics from being eligible to volunteer with medical humanitarian organizations.

With degree-education and state regulation, paramedics in the UK have an enviable position as autonomous healthcare professionals, shared only with paramedics in Australia and New Zealand^45^ and South Africa.^46^ While paramedicine exists in other countries across the world, Freidson^35^ would undoubtably argue that the lack of self-governance afforded to the occupation in some of these countries (either through lack of autonomy, as in the Franco-German model, or lack of education as in the Anglo-American model) fail to make it a profession *suo jure*. This again raises issues of professional identity regarding what a paramedic *is* and what it means to *be* a paramedic.^37^ However, whether this causal sequence holds or not remains a matter for further discourse and research consideration.

## Summary

In the United Kingdom, the transformation of the paramedic profession over the last decade has made paramedics ideal for transference across other clinical practice settings. Educated to degree level with autonomous registration, pluripotent by design and generalist through their experiences in ambulance services, there are increasing opportunities for paramedics to work across a variety of clinical settings. However, it is during this movement that concerns arise due to diversification away from what is assumed of paramedic professional identity. These concerns are heightened by the lack of knowledge and awareness of the profession by other healthcare professionals; under-representation in organizations where they are not the dominant profession; failure to accurately capture the workforce in census data; and a lack of visibility in policy and within leadership in positions. For the paramedic profession to continue to develop throughout the 21st Century, these issues need to be addressed in order for them to retain their position and continue to demonstrate their value as part of the healthcare workforce.

## Data Availability

No new data were generated or analyzed in support of this review.
